# Nursing diagnoses in adult/elderly patients undergoing outpatient antineoplastic chemotherapy: a review

**DOI:** 10.3332/ecancer.2017.736

**Published:** 2017-05-03

**Authors:** Rafael Tavares Jomar, Rebeca Anselmo Furtado Gomes, Dayana Carvalho Leite, Helena Ferraz Gomes, Ellen Marcia Peres, Eugenio Fuentes Perez Junior

**Affiliations:** 1 Faculty of Nursing, Rio de Janeiro State University, Rio de Janeiro, RJ 20551-030, Brazil; 2 Pedro Ernesto University Hospital, Rio de Janeiro State University, Rio de Janeiro, RJ 20551-030, Brazil

**Keywords:** oncology nursing, nursing process, nursing diagnosis, drug therapy/antineoplastic agents, ambulatory care

## Abstract

**Objective:**

To search in the scientific literature for nursing diagnoses identified in adult/elderly patients undergoing antineoplastic chemotherapy in an outpatient setting.

**Methods:**

Review of studies published in Portuguese, English, or Spanish which were searched in five electronic databases in March 2016, using the descriptors nursing process, nursing diagnosis, neoplasms, drug therapy and hospital outpatient clinic.

**Results:**

In the four studies selected for review, 40 nursing diagnoses were identified, namely unbalanced nutrition, less than body requirements, risk of deficient fluid volume, diarrhoea, fatigue, impaired home maintenance, deficient knowledge, disturbed body image, interrupted family processes, ineffective sexuality pattern, anxiety, powerlessness, fear, readiness for enhanced religiosity, risk of infection, impaired dentition, risk of impaired skin integrity, acute pain, and nausea.

**Conclusions:**

The nursing diagnoses identified can support the selection of interventions and the creation of nursing guidelines in outpatient oncology services.

## Introduction

The present study deals with the diagnostic stage of the nursing process. Defined as a clinical judgment based on the data collected in the nursing history, culminating in decision making about the diagnostic concepts that represent the responses of the person, family or community at a given moment in the health-disease process, nursing diagnosis (ND) is the basis for the selection of interventions aimed at achieving results for which the nurse is responsible [1].

According to Resolution No. 358/2009 of the Federal Nursing Council, the disciplinary body for professional nursing practice in Brazil, the nursing process must be carried out deliberately and systematically in all public and private environments in which professional nursing care takes place. Therefore, it should be formally documented in order to highlight the contribution of nursing to the health care of the population and, consequently, to increase visibility and professional recognition [2].

In oncology services, the nursing process is imperative due to the high frequency of physical and psychological damage that compromises patients’ quality of life [3]. This is because the treatment modalities for cancer, especially chemotherapy, usually present toxic effects such as myelosuppression, alopecia, fatigue, nausea, vomiting and diarrhoea [4]. Therefore, nurses have a great responsibility for the planning of nursing care in oncology, especially regarding decision making and actions aimed at solving problems identified in the diagnostic stage [3].

The estimation for Brazil for the years 2016–2017 [5] indicates the occurrence of about 600,000 new cases of cancer. Excluding non-melanoma skin cancer (approximately 180,000 new cases), there will be about 420,000 new cases of cancer, where prostate (61,000) and breast (58,000) cancers will be the most common. After non-melanoma skin cancers, the most frequent types of cancers in men will be prostate (28.6%), lung (8.1%), intestinal (7.8%), stomach (6.0%), and oral (5.2%). In women, breast (28.1%), intestinal (8.6%), cervical (7.9%), lung (5.3%), and stomach (3.7%) cancers will be among the main types.

Given this estimate and knowing that cancer patients are frequently treated with antineoplastic outpatient chemotherapy, nurses working in oncology outpatient services should provide care with a focus on the individual’s needs, using the ND as a tool in the context of nursing process for selecting interventions with the goal of improving patients’ responses to the proposed antineoplastic treatment. Considering the lack of publications on ND in oncology and the fact that knowledge of common NDs in this area can strengthen the nursing process, in addition to providing oncology nurses with support for decision-making, choosing the best interventions, and performing a quality clinical practice, the objective of this study was to search in the scientific literature for NDs identified in adults/seniors undergoing antineoplastic chemotherapy in an outpatient setting.

## Methods

This is a review of the scientific literature which involved gathering, analysing and synthesising, in a systematic and orderly way, results of studies that investigated NDs in adults and seniors undergoing outpatient chemotherapy according to the following steps: formulation of the guiding question, search of the study literature, interpretation and synthesis of the evidence from the selected studies. The guiding question for this review was the following: Which nursing diagnoses have already been identified in adults/seniors undergoing anticancer chemotherapy in an outpatient setting?

In March 2016, with no restrictions on the nature of the study or language, two authors independently searched for indexed studies in the electronic databases *Medical Literature Analysis and Retrieval System Online* (MEDLINE), *Cumulative Index to Nursing and Allied Health* (CINAHL), Latin American and Caribbean Literature in Health Sciences (LILACS), *Scientific Electronic Library Online* (SciELO), and Nursing Database (BDENF). As a search strategy, 12 different combinations of the descriptors were used: Nursing Process (*Nursing Process*), Nursing Diagnosis (*Nursing Diagnosis*), Neoplasms (*Neoplasms*), Chemotherapy (*Drug Therapy*) and Outpatients Clinics Hospital (*Outpatient Clinics Hospital*) (Table 1). The selection of descriptors used in the search process of the studies was done through consultation of the DeCs (BIREME Descriptors in Health Sciences) and MeSH (Pubmed *Medical Subject Headings*).

The criteria for inclusion of studies in the review were as follows: be published in original article format in English, Spanish, or Portuguese; having as study subjects adult and/or elderly individuals undergoing antineoplastic chemotherapy on an outpatient basis and; the NDs being identified in this population, according to *NANDA International* [1] Taxonomies I or II. The exclusion criteria adopted were literature reviews, updates, experience reports or any other format where it was not stated that the results were obtained from collection of data from adults/seniors undergoing outpatient chemotherapy (interview and/or physical examination) or review of their medical records. It should be noted that the eligibility criteria adopted were based on the fact that the main evidence supporting clinical practice comes from research results [6].

5,941 studies were found in the MEDLINE database, in addition to 186 in CINAHL, 227 in LILACS 227, 152 in BDENF, and 17 in SciELO, for a total of 6,523 studies, which were then preselected from those with titles and abstracts which met the criteria explained previously. In the MEDLINE database, two studies were preselected, in CINAHL nine, in LILACS eight, in BDENF three, and SciELO none, for a total of 22 studies. After elimination of duplicates and reading of the studies in full, the final sample of studies selected for the present review consisted of four original papers [7–10]. The references cited by the selected articles were analysed to identify publications not found by the search strategy in the electronic databases, but no additional studies were identified at this stage. The process described earlier was performed by the same authors who searched for studies and any disagreements were resolved through consultation with a third author.

In order to extract information from the articles selected for the review, a data collection form was developed in which all NDs identified by each of the selected articles were listed. The form also allowed us to obtain information on the authors, country, and year of publication of the article, with objectives, design, data source, sample type, conclusions and recommendations for nursing practice. The interpretation and synthesis of the evidence gathered from the selected articles was done in a descriptive manner, allowing the reader to understand each study included in the review, and considering the quality criteria established by nursing students [11].

## Results

A summary of the main features of the articles included in the review is presented in Table 2. Among the countries of origin of the studies, Brazil stood out with three of the four articles selected [7–8, 10], of which only one was not published in Portuguese, but in Spanish [9]. Regarding the year of publication, three articles were published between 2008 and 2010 [8–10]. As for the data source, only one article used the patient’s chart in addition to the interview and physical examination [8]. In relation to *NANDA International* [1] Taxonomies, only one article did not specify which Taxonomy was used [7], while all others used Taxonomy II. The sample size of the studies ranged from 10 to 90 individuals, whose ages ranged from 22 to 89 years. Regarding the type of sample, all studies had non-probabilistic samples, that is, samples of convenience.

As all studies selected for the review are descriptive, the low level of quality of the evidence generated by them is highlighted: level 6, on a scale of 1 to 7, where level 1 represents the highest quality Evidence [11].

Of the 34 actual NDs and six risks identified, at least half of the component articles in the review described the following: unbalanced nutrition, less than body requirements, risk of deficient fluid volume, diarrhoea, fatigue, impaired home maintenance, deficient knowledge, disturbed body image, interrupted family processes, ineffective sexuality pattern, anxiety, powerlessness, fear, readiness for enhanced religiosity, risk of infection, impaired dentition, risk of impaired skin integrity, acute pain, and nausea (Table 3).

## Discussion

In order to provide an overview of the most common NDs in adults/seniors undergoing outpatient chemotherapy and thus to provide support for the decision-making of the oncology nurse during the completion of the nursing process, the present review identified 40 ND: 34 real and six at risk. Only in the growth/development domain, no NDs were identified, which was expected, as in this domain there are only NDs related to age-appropriate development (childhood and adolescence) [1].

All articles selected have described the ND body image disorder, defined as confusion in the mental image of the physical self [1]. Antineoplastic chemotherapy was indicated by a review study on body image in cancer patients [12] as one of the factors responsible for the occurrence of this ND is due to one of its most common side effects: alopecia. This is of greater importance among women who have had a mastectomy and individuals with intestinal stomas who undergo antineoplastic chemotherapy, as reported in articles that are part of this review [7–8, 10].

Only one selected article [10] did not identify the fatigue ND. Defined as an oppressive and prolonged feeling of exhaustion and diminished ability to perform physical and mental work at the usual level [1], fatigue is one of the most prevalent symptoms in cancer patients, being reported by 50–90% of patients during the course of the disease or its treatment [13]. Therefore, cancer patients should be investigated for fatigue whenever they are submitted to antineoplastic chemotherapy so that pharmacological and non-pharmacological measures are adopted as soon as it is identified [14].

The ND of anxiety was described by almost all the articles components of this review [7–8, 10]. Defined as a vague and uncomfortable feeling of discomfort or fear, of apprehension caused by the anticipation of danger, the anxiety disorder is a warning sign that draws attention to an imminent danger and allows the individual to take action to deal with the threat [1]. A study that evaluated the perception of anxiety in patients submitted to antineoplastic chemotherapy pointed out the side effects of this therapy as one of the main factors responsible for this feeling [15].

An experimental study that evaluated the effect of prayer on the anxiety of cancer patients in chemotherapy treatment [16] showed that prayer is an efficient strategy to reduce this feeling. As the readiness for enhanced religiosity—defined as a pattern of trust in religious beliefs and/or participation in rituals of a religious faith that can be strengthened [1]—has been described by half of the articles selected for this review [9–10], the nursing team could use prayer as a strategy to provide spiritual support to cancer patients on chemotherapy, in order to meet the needs of their spirituality as well as to help them control anxiety [16].

Defined as a response to the perceived threat that is consciously recognised as a hazard, the ND Fear [1] was also described by half of the review articles [8–9]. The social stigma of cancer may lead patients to experience feelings of dread in the face of disease [8]. A validation study of the content of the defining characteristics of NDs anxiety and fear [17] emphasised the difficult differentiation of these two diagnoses. In addition to their defining characteristics being similar and mostly subjective, these NDs are often presented concomitantly by the same patient, as observed in a selected article to compose the present review [8].

In view of this, it is important to note that anxiety and fear are among the defining characteristics of ND. Defined as a perception of lack of comfort, relief and transcendence in the physical, psychospiritual, environmental, cultural and/or social dimensions, the ND acute pain [1] was identified by half of the review articles [9–10]. In addition to those highlighted, other defining characteristics of this ND, such as treatment regimen and symptoms related to the disease, help to explain its occurrence in adults/elderly subjects submitted to antineoplastic chemotherapy. In addition to the pain that can be generated by the tumour itself, this therapy can cause several painful side effects such as headache, myalgia, gastralgia, burning and tingling [4].

Nausea and diarrhoea are frequent toxic effects caused by antineoplastic chemotherapy [4]. Diarrhoea, defined as the elimination of loose and unformed stools [1], and nausea, defined as a subjective phenomenon of unpleasant sensation in the back of the throat and stomach, which may or may not result in vomiting [1] , were also identified by half of the studies selected to compose the review [7, 9], such as the unbalanced nutrition ND: lower than the body needs [7–8], whose definition is insufficient intake of nutrients to satisfy the metabolic needs [1].

NDs nausea and unbalanced nutrition: less than bodily needs have a defining characteristic in common, food aversion [1]. A hypothesis that helps to explain the occurrence of unbalanced nutrition ND: less than body requirements in adults/seniors submitted to outpatient antineoplastic chemotherapy is the nausea caused by this modality of cancer treatment, which, in turn, can provoke a feeling of vomiting and aversion to food, thus leading the individual to insufficient intake of nutrients to satisfy his metabolic needs. An integrative review on ND nausea, concluded that young age, female gender, motion sickness, emetic potential of chemotherapy, anxiety, conditioned stimulus and expectation of nausea after treatment are the most frequent factors related to the occurrence of this condition in patients submitted to antineoplastic chemotherapy [18].

It is worth noted that diarrhoea is also one of the defining characteristics of unbalanced nutrition ND: less than body requirements [1]. Increasing the frequency of loose stools, unformed or liquid, can impair the permanence of food ingested in the gastrointestinal system long enough for absorption of different nutrients to occur properly [19].

The impaired dentition ND, defined as rupture in dental developmental/eruption patterns or structural integrity of each tooth [1], was identified by half of the studies selected for review [9–10]. Such an ND has absence, wear, looseness and tooth decay as some of its defining characteristics and inadequate oral hygiene as one of its related factors [1]. Considering that patients submitted to antineoplastic chemotherapy have risk factors for oral and systemic alterations and that adequate oral hygiene is determinant of overall health, the nurse must act in the teaching of hygiene and supervision of the oral conditions of patients submitted to this therapy [20].

A selected study [8] for this review states that special attention should be given to the impaired home maintenance ND whose definition is the inability to independently maintain a safe environment for growth promotion [1]. This is because the submission to antineoplastic chemotherapy can exclude the patient from the social roles usually performed, making him feel powerless in the face of this cancer treatment modality [8], which often has an impact on the maintenance capacity of the home due to its toxic effects.

Powerlessness is an ND that is defined by the lived experience of lack of control over a situation, including a perception that the actions themselves do not significantly affect a result [1]. One of its defining characteristics stands out: frustration about the inability to perform normal activities [1]. This inability—momentary, it is worth emphasising—is probably due to the toxic effects of antineoplastic chemotherapy, such as fatigue, one of the most prevalent symptoms in cancer patients undergoing treatment [13].

The ND Interrupted family process, defined as change in relationships and/or family functioning [1], was described by two studies selected for review [7, 9]. It is possible that the change in the health status of a family member [1]—one of its related factors—is mainly responsible for its occurrence in adults/elderly patients submitted to outpatient antineoplastic chemotherapy.

The ND pattern of ineffective sexuality, defined as an expression of concern about one’s own sexuality, was also described by two studies selected to compose the review [7, 9]. These studies were conducted mainly in women with breast cancer submitted to antineoplastic chemotherapy, which helps explain the identification of this ND, since, especially among mastectomised women, body image affects sexuality [21].

The ND deficient knowledge, defined as absence or deficiency of cognitive information related to a specific topic [1], was identified by half of the review studies [7, 10]. However, only one of them specified the topic with poor information (disease and treatment process) [7]. It is worth mentioning that nurses can play an important role as educators by providing information on cancer, antineoplastic chemotherapy, and its potential complications in order to answer questions and promote the health of patients under treatment.

In the present review, six of the 40 nursing diagnoses identified were at risk. The fact that the majority of NDs are real indicates that nursing care for adults/seniors undergoing outpatient anticancer chemotherapy is focused on health recovery. On the other hand, the identification of risk NDs also suggests that nurses are concerned about preventive aspects of care.

Defined as vulnerability to invasion and multiplication of pathogenic organisms, which may compromise health, risk of infection [1] was identified by half of the articles reviewed [8–9]. This is one of the major NDs faced by oncology nurses due to the great predisposition of patients undergoing antineoplastic chemotherapy to contract infections, since this modality of cancer treatment can cause immunosuppression [4, 22].

Both ND risk of impaired skin integrity, defined as vulnerability to alteration in the epidermis and/or dermis, and ND deficient fluid volume, defined as vulnerability to decreased intravascular, interstitial and/or intracellular fluid, have pharmacological agents as a related risk factor [1]. It is known that many chemotherapy drugs can cause hypersensitivity, dryness and desquamation of the skin, as well as require the administration of large volumes of intravascular physiological solution to avoid potential complications [22].

As all the studies selected for this review have a descriptive character, we must highlight the low level of the quality of the evidence generated by them [11]. However, it is worth emphasising that descriptive observational studies are inevitably the most likely to achieve their objectives [23]. Although the isolated results of the studies that compose this review do not constitute irrefutable recommendations, we emphasise that the combination and synthesis of them—done here—can support the selection of interventions and the elaboration of nursing guidelines in outpatient oncology services.

Among the limitations of this review we point out the search and selection of studies in the form of a research article indexed in electronic databases only and the different characteristics of the populations of the studies that comprise it: two were conducted on women with breast cancer [10], one on people with colorectal cancer [8] and the other on people undergoing chemotherapy treatment for several types of cancer [9]. However, although the characteristics of the patients and the cancer being treated are different, the adults/subjects participating in the studies have in common the fact that they underwent anticancer chemotherapy, which allows for some generalisation of our findings to similar populations.

## Conclusions

Given the scarcity of publications on the diagnostic phase of the oncology nursing process, the 40 NDs identified by the present review point to the complexity of the care provided to patients undergoing chemotherapy in an outpatient setting. If used as a guide for the investigation of NDs among adult/elderly users of oncology outpatient services, the results of this review may contribute to the advancement and improvement of the nursing process.

## Conflicts of interest

The authors declare that there are no conflicts of interest.

## Authors’ contributions

RTJ participated in the design and outline of the article, bibliographic research, data analysis and interpretation, discussion of results, writing, and final approval of the article.

RAFG participated in the bibliographic research, data analysis and interpretation, critical review, and final approval of the article.

DCL participated in the bibliographic research, critical review, and final approval of the article.

HFG, EMP, and EFPJ participated in the critical review and final approval of the article.

## Figures and Tables

**Table 1.** Combinations of descriptors used as a search strategy for the studies.

1. *Nursing Process* AND *Neoplasms* AND *Drug Therapy* AND *Outpatient Clinics Hospital*
2. *Nursing Diagnosis* AND* Neoplasms* AND* Drug Therapy* AND* Outpatient Clinics Hospital*
3. *Nursing Process* AND* Neoplasms* AND* Drug Therapy*
4. *Nursing Diagnosis* AND* Neoplasms* AND* Drug Therapy*
5. *Nursing Process* AND* Neoplasms*
6. *Nursing Diagnosis* AND* Neoplasms*
7. *Nursing Process* AND* Drug Therapy*
8. *Nursing Diagnosis* AND* Drug Therapy*
9. *Nursing Process* AND* Drug Therapy* AND* Outpatient Clinics Hospital*
10. *Nursing Diagnosis* AND* Drug Therapy* AND* Outpatient Clinics Hospital*
11. *Nursing Process* AND* Neoplasms* AND* Outpatient Clinics Hospital*
12. *Nursing Diagnosis* AND* Neoplasms* AND* Outpatient Clinics Hospital*

**Table 2.** Main characteristics of the component studies of the review

Author/country/year	Design/source/sample	Objective
Narchi and Gutiérrez, Brazil, 1997 [7]	Descriptive study Interview Non-probabilistic (*N* = 14)	To identify the defining characteristics of nursing diagnoses found in women with breast cancer who are undergoing outpatient antineoplastic chemotherapy
Silva and Gorini, Brazil, 2008 [8]	Case studies Interview, physical examination, and patient chart Non-probabilistic (*N* = 11)	To identify nursing diagnoses in patients with colorectal cancer who are undergoing outpatient antineoplastic chemotherapy
Manrique and Pedraza, Colombia, 2008 [9]	Cross-sectional study Interview and physical examination Non-probabilistic (*N* = 90)	To estimate the prevalence of nursing diagnoses in patients undergoing outpatient antineoplastic chemotherapy
Oliveira *et al*, Brazil, 2010 [10]	Descriptive study Interview and physical examination Non-probabilistic (*N* = 10)	To describe the application of nursing care systematisation in women who have had a mastectomy and are undergoing outpatient antineoplastic chemotherapy

**Table 3.** Nursing diagnoses in adults/elderly undergoing outpatient antineoplastic chemotherapy, according to domain and class (*n*/*N*)*.

**Domain 1. Health promotion** **Class 1. Health awareness** Deficient diversional activity (1/4) Sedentary lifestyle (1/4) **Class 2. Health management** Readiness for enhanced health management (1/4)
**Domain 2. Nutrition** **Class 1. Ingestion** Unbalanced nutrition: less than body requirements (2/4) **Class 5. Hydration** Risk of deficient fluid volume (2/4)
**Domain 3. Elimination and exchange** **Class 1. Urinary function** Impaired urinary elimination (1/4) **Class 2. Gastrointestinal function** Constipation (1/4) Risk of constipation (1/4) Diarrhoea (2/4)
**Domain 4. Activity/rest** **Class 1. Sleep/Rest** Disturbed sleep pattern (1/4) **Class 3. Energy balance** Fatigue (3/4) **Class 5. Self-care** Bathing self-care deficit (1/4) Impaired home maintenance (2/4)
**Domain 5. Perception/cognition** **Class 4. Cognition** Deficient knowledge (2/4) Readiness for enhanced knowledge (1/4) **Class 5. Communication** Readiness for enhanced communication (1/4)
**Domain 6. Self-perception** **Class 1. Self-concept** Hopelessness (1/4) **Class 2. Self-esteem** Situational low self-esteem (1/4) Risk for situational low self-esteem (1/4) **Class 3. Body Image** Disturbed body image (4/4)
**Domain 7. Role relationships** **Class 2. Family relationships** Interrupted family processes (2/4) **Class 3. Role performance** Impaired social interaction (1/4)
**Domain 8. Sexuality** **Class 2. Sexual function** Ineffective sexuality pattern (2/4)
**Domain 9. Coping/stress tolerance** **Class 2. Coping responses** Anxiety (3/4) Powerlessness (2/4) Fear (2/4) Impaired resilience (1/4)
**Domain 10. Life principles** **Class 2. Beliefs** Readiness for enhanced spiritual well-being (1/4) **Class 3. Value/belief/action congruence** Readiness for enhanced religiosity (2/4) Spiritual distress (1/4)
**Domain 11. Safety/protection** **Class 1. Infection** Risk for infection (2/4) **Class 2. Physical injury** Impaired dentition (2/4) Impaired skin integrity (1/4) Risk of impaired skin integrity (2/4) Impaired tissue integrity (1/4) Risk for injury (1/4)
**Domain 12. Comfort** **Class 1. Physical comfort** Impaired comfort (1/4) Acute pain (2/4) Nausea (2/4) **Class 3. Social comfort** Social isolation (1/4)
**Domain 13. Growth/development** The articles selected did not identify nursing diagnoses in this Domain


* (*n*/*N*): *n* = number of articles that identified the nursing diagnosis; *N* = 4 review component articles.
